# The Seven Selves of Dementia

**DOI:** 10.3389/fpsyt.2021.646050

**Published:** 2021-05-14

**Authors:** Iris Bomilcar, Elodie Bertrand, Robin G. Morris, Daniel C. Mograbi

**Affiliations:** ^1^Institute of Psychiatry, Federal University of Rio de Janeiro, Rio de Janeiro, Brazil; ^2^Laboratoire Mémoire, Cerveau et Cognition (LMC2, URP 7536), Institut de Psychologie, Université de Paris, Paris, France; ^3^Department of Psychology, King's College Institute of Psychiatry, Psychology and Neuroscience, London, United Kingdom; ^4^Department of Psychology, Pontifical Catholic University of Rio de Janeiro, Rio de Janeiro, Brazil

**Keywords:** dementia, Alzheimer's disease, selfhood, self, awareness

## Abstract

The self is a complex and multifaceted phenomenon, encompassing a variety of cognitive processes and psychosocial influences. Considering this, there is a multiplicity of “selves,” the current review suggesting that seven fundamental self-processes can be identified that further our understanding of the experience of dementia. These include (1) an embodied self, manifest as corporeal awareness; (2) an agentic self, related to being an agent and influencing life circumstances; (3) an implicit self, linked to non-conscious self-processing; (4) a critical self, which defines the core of self-identity; (5) a surrogate self, based on third-person perspective information; (6) an extended self, including external objects or existences that are incorporated into the self; and, finally, (7) an emergent self, a property of the self-processes that give rise to the sense of a unified self. These are discussed in relation to self-awareness and their use in making sense of the experience of dementia.

## Introduction

Despite the elusiveness of the concept, most of us can apprehend the notion of the self. In everyday life, our access to the world happens through a first-person perspective. Philosophically, approaches to understanding the self have been developing, a main one being that the self is ontologically subjective (1); for example, in his philosophy of mind, William James suggesting that feelings and thoughts only exist in relation to an experiencer of the experience (1, 2). Searle (1) defines this as a fundamental property of consciousness, namely, first-person ontology. The first-person perspective of most of our experiences attest, from a phenomenological point of view, the idea of an underlying construct that modulates our apprehension of the world: the self.

Further evidence for this property of our minds is seen by the plethora of alterations in self-experience caused by neurological and psychiatric conditions. The range and variety of these self-changes indicate that these phenomena are underpinned by multiple neural processes, with different aspects of the self being linked to the activity of specific brain regions. It is important to highlight that, when discussing the self, we are referring to processes and not to something inside our brains. This avoids reifying the concept, which could lead to a homunculus fallacy (3). Similarly, as it will be seen in the next sections, these self-processes extend through different levels of complexity. This is a crucial point, indicating that there is no circularity or infinite regress.

Indeed, the experience of the self is the likely interaction of a multiplicity of self-processes. Different brain networks, in interaction with the environment, including interpersonal values and cultural norms, lead to diverse self-related abilities, such as bodily awareness, agency, and autobiographical memory, amongst others (4). It has been suggested that these different forms of self may have a hierarchical relationship, with some self-processes establishing themselves earlier on and providing the scaffolding for higher-order processes (5). In this nested hierarchy approach, certain aspects of the self have a central position, responding to essential processes that allow the emergence of other complex self phenomena (5).

Exploring the processes that concern the “self” in dementia has many important consequences. Considering the progressive neurodegenerative characteristic of dementia, relatives and caregivers may struggle to see beyond the condition as a diagnosis and disease state. This may lead to caring practices that diminish personhood in dementia, which has been termed “malignant social psychology” (6). Institutional and individual practices with depersonalizing elements not only disempower and stigmatize people with dementia (PwD) but also undermine them, potentially causing excess disability (7).

Investigating how self-processes are affected by dementia may support humanizing care practices, relying on preserved abilities and promoting personhood in the condition. However, for this to happen effectively, an appreciation of the impact of dementia-related impairments is needed. Only by knowing which aspects of the self are more resilient to change and which ones are vulnerable to the losses caused by dementia can we achieve better standards of care for PwD and their families.

Considering this, the current article aims to discuss how self-processes can be impacted by dementia. Specifically, seven forms of the self will be explored: embodied, agentic, critical, implicit, surrogate, extended, and emergent. These seven aspects of the self were selected as main constructs that explain the totality of the self and that have been considered individually in relation to PwD, also taking into account clinical phenomena; the final concept of self, namely, “the emergent self,” was generated to incorporate the missing notion that the different aspects may interact to form emergent properties, the sum being greater than the constituent parts. These aspects of self will be discussed in relation to existing evidence, from both a cognitive neuroscience approach and a social psychology/ethnographic approach. Future research directions and the clinical implications of these forms of self are explored in the conclusion.

## Seven Forms of the Self in Dementia

### Embodied Self

Accumulating evidence in the literature suggests that our body plays an essential role in the constitution of a sense of self (5). In this context, the body is not seen as a part of the physical world but as a vehicle that allows us to be a self in this world (8–10). Some researchers attribute the embodied self to bodily capacities, such as multisensory integration, interoception, and agency (11), which occur at an implicit level and emerge as a unified experience of the body via perception and action (12–14). Considering the various aspects of the embodied self, studies suggested that its neural correlates involve posterior brain regions, particularly the temporoparietal junction and the extrastriate body area (15, 16). In a recent resting-state functional connectivity study, Piras et al. (17) suggest that the activity of the brain at rest is influencing individual self-attribution by linking somatosensory representations to visual and proprioceptive information (17). Moreover, the embodied self is described to be essential in the foundation of other kinds of selves, which rely on higher levels of brain function (5, 18). In fact, it has been argued, in the context of dementia, that bodily processes may provide the basis for structuring or preserving some cognitive abilities (19).

Considerable evidence has been generated indicating the endurance of the self in PwD, despite cognitive decline. For example, Kontos (20), in an ethnographic study, demonstrates that aspects of the self may persist in people with severe dementia. A potential explanation for these findings is that the embodied dimension of human existence is, as suggested above, grounded ontologically on implicit processes (21), with the embodied self preceding and not having to emerge from cognitive forms of knowledge (20). Such processes are more resilient in relation to neurodegeneration, being structured by interaction with bodily control. This embodied dimension may manifest itself through action, such as dancing, caring, and gestural communication, referring to the complex inter-relationship between corporeal (i.e., physical movement) and social characteristics of the body (i.e., the social and personal meaning of the movement) [(20, 21), for a review, (22)].

One important feature of the embodied self refers to our gestures during communication (i.e., facial expression, gaze, movements of the body, intonation, and tone of voice), which are considered a form of natural expression of the body (8). Gestures are an important topic when discussing dementia, as they provide a different perspective on how PwD communicate. It has been shown that PwD who have lost language abilities are still able to communicate non-verbally, through eye gaze, facial expressions, and movement (23, 24). In relation to facial expressions, although PwD have shown impairments in recognition, a number of studies have suggested preserved, albeit attenuated, facial reactivity to emotional stimuli, such as films (25), computer tasks (26), and faces (27). In these studies, responses of participants were coherent with the emotional stimuli, suggesting preserved bodily processes that are present even in severe stages of the condition [e.g., (27)]. Similarly, reactivity to pain stimuli with facial expressions has also been shown across severity levels in dementia (28).

Another example of the embodied self refers to artistic expression and creativity by drawing, which is often maintained in dementia. There are descriptions in the literature of people in later stages of Alzheimer's disease (AD) with severe cognitive impairments, who, nevertheless, preserve the ability to draw (29, 30). In this case, the longer-lasting persistence of creativity is associated with the embodied self, as it represents the expressiveness of the body, despite or beyond cognition. In other words, the body is capable of learning and transforming its perception into something meaningful and symbolic (31).

An important topic when considering the embodied self in PwD is appearance (20). For instance, some authors have suggested the importance of maintaining gendered appearance (i.e., haircut, clothing, and cosmetics) as a form of support for the self in dementia (32–34). Gove (35) argues that one of the critical factors that lead to stigmatization is when PwD appearance is not considered adequate in certain social contexts. Given the loss of autonomy for self-care (e.g., bathing and dressing) in later stages of dementia, this may lead to alienating PwD in relation to their appearance, with family members or caregivers assuming these responsibilities and adopting their own way or institutional norms to conduct these activities (35). Considering this, some authors have suggested that, more important than the final result, the process used to take care of PwD appearance is a crucial factor in structuring the self at this level (36, 37).

While the embodied self is shown to have levels of preservation in dementia, there is evidence that interoceptive processing (i.e., awareness of visceral states) is impaired in the condition. In a neuroimaging study (38), people with AD (PwAD) and behavioral-variant frontotemporal dementia (bvFTD) showed resting-state functional connectivity alterations in specific hubs of the fronto-temporo-insular network, which were associated with impairment on different interoceptive dimensions (accuracy, learning, and awareness). Both patient groups showed reduced interoceptive awareness, overestimating their ability to detect interoceptive changes, with changes in heartbeat and learning using stethoscopic feedback (interoceptive learning). The AD group exhibited a differential deficit in such learning, arguably linked to memory dysfunction. Other interoceptive disruptions, particularly associated with altered pain and temperature processing, were also reported in bvFTD, with both hyper- and hypo-responsiveness observed (39). These disruptions were associated with gray matter volume of the right mid and posterior insula (39, 40). Studies exploring the neural correlates of interoceptive awareness in healthy population highlight similar networks, including specifically the insular cortex (41, 42).

### Agentic Self

The experience of controlling one's own actions and, through them, influencing the course of external events has been termed “sense of agency” (43). A central aspect of this phenomenon involves the subjective experience of a voluntary action, including the intend to act, the choice between possible actions, and the initiation of these actions, which relies essentially on cognitive processes (44, 45). An additional aspect involved in the sense of agency is experiences related to the feeling of ownership subjacent to the activity of peripheral somatosensory receptors while the body is moving, also termed volition (43). Finally, the sense of agency includes the association between the subjective experience of one's own action and the observed changes in the external world caused by one's own action.

Agency is incorporated into neurological models of anosognosia, or awareness of deficit, such as the feedforward intentional model by Heilman, Barrett, and Adair (46) in which signals relating to intention to act are compared with kinesthetic action signals to regulate awareness of bodily action, also providing a sense of agency. The neural correlates subjacent to the sense of agency are still unclear; nevertheless, evidence suggests that agency may rely on the connectivity between frontal and prefrontal motor areas involving the initiation of movements and the parietal cortex supporting the monitoring of perceptual events [for a review, see (43)]. In addition, new insights from neuroimaging studies highlighted the role of resting-state activity in regions responsible for preparing information for visuo-motor integration. Specifically, it has been suggested that the activity of the brain at rest in action-related perceptual and motor circuits needs to interact in order to create a feeling of control over events (17).

In a social psychology perspective, agency is understood as people proactively participating in their development, adaptation, and actualization of self-knowledge, following changes over time (47–49). Bandura (49) describes three core features of human agency: (1) forethought, which is related to the adaptation of self-interest using past experiences in order to provide direction, coherence, and meaning to one's life and involving the capacity of anticipation and visualization of goals and outcomes to motivate efforts; (2) self-reactiveness, linked to self-regulation and motivation of actions during the execution of a plan; and (3) self-reflectiveness, which is associated with the role of people not only as agents of actions but also as self-examiners of their own functioning, including evaluation of performance, integrity and meaning of thoughts and actions, and adjustment to possible errors.

Agency is normally explored focusing on decision-making and self-reflective capacities, which are directly linked to different cognitive abilities, including language, deliberative thoughts, and intentional and goal-oriented actions (49, 50). For example, the dissociable interactions and conscious experience model by Schacter (51) considers that interaction between basic cognitive abilities is mediated by consciousness. The literature suggests that a combined deficit in decision making and self-reflection in PwD may result in diminished sense of agency (52). A more extensive concept of agency might be needed considering the experiences of agency in people with cognitive difficulties. Models such as those proposed by Kontos (53), based on a phenomenological perspective, respond to this demand, understanding agency in PwD as originating principally from bodily information, action, and goal-directed behavior (44, 45). Additionally, acknowledging the socio-emotional, in addition to the cognitive, basis of reflexivity would enable the recognition of agency in PwD, even in severe stages of the condition (54, 55). Current findings support this notion. For example, Boyle (56) explored the potential for agency in people with mild and moderate dementia, and results showed that while participants present with difficulties in decision-making capacity, they were able, however, to demonstrate a sense of agency through behavioral and emotional responses (56).

Another important aspect discussed in the literature refers to a possible relation between agency and autonomy (57). In the context of impairments in decision making in dementia, few possibilities are offered to PwD in their daily life to make choices and, therefore, to practice and maintain a sense of agency and autonomy. In a systematic review exploring the experience of agency in PwD, Bosco et al. (57) described three different decision-making pathways, reflecting the degree of autonomy and agency in this group: decision making, shared decision making, and pseudo decision making. Each of these pathways has a different impact on the agency of PwD, encouraging or discouraging decision-making practices (57).

A few studies explored social interferences in human agency in the context of dementia. For example, Boyle and Warren (55) investigated whether people with severe dementia demonstrate emotional reflexivity within their personal relationships (i.e., PwD and their spouses). The authors observed that, even when PwD abilities for deliberation, discourse, and social interaction were limited, they could nevertheless demonstrate emotional reflexive abilities necessary to exercise agency within their daily lives. This suggests that PwD might express their selves using mechanisms other than verbal language, such as perception, feelings, and desires in habituated, embodied, and emotional forms (55). Furthermore, Chung et al. highlighted the strategies adopted by carers that encourage and sustain a sense of autonomy and agency in PwD, reinforcing the importance of social factors for agency in this group (58).

In conclusion, the findings emphasize the relevance of assisted autonomy in dementia, including support for practicing and maintaining a sense of agency, which is closely linked to personhood in PwD (59). Such practices allow patients to engage in meaningful activities, with individuals gaining a sense of choice, commitment, positive meaning, and interaction (60). By contrast, it has been shown that lack of such support can lead to behavioral and psychological symptoms, including restlessness, wandering, depression, and agitation (58).

### Implicit Self

Damasio suggests that part of the information that grounds our sense of self exist in implicit states (18). This is in accordance with research on social cognition, which indicates the existence of knowledge about the self, in forms inaccessible for consciousness (e.g., unaware thoughts and emotions) but which can be tapped indirectly (61). Moreover, there is a substantial body of clinical and experimental evidence that shows that behavioral and affective responses can be influenced by implicit information processing in a variety of neurological conditions [for a review, (62)]. In this context, the implicit self is related to responses that suggest self representations, preferences, and attitudes that are implicit in nature. The neural correlates of implicit self-awareness are still unclear in the literature; however, studies exploring this phenomenon with neurological patients suggest that implicit processing involves more subcortical regions, such as the basal ganglia and the amygdala, compared with explicit processing, supporting more cortical regions (63, 64). In line with these neural findings, authors suggested the existence of parallel routes, implicit and explicit cognitive mechanisms, for processing of similar information (62, 65, 66).

PwD often lack awareness about their condition and associated changes, neurologically referenced as anosognosia (67). Awareness in PwD is often assessed by contrasting self-report, at an explicit level, with clinician/informant opinion or performance in tasks (68). Nevertheless, it has long been noted that despite explicit unawareness, PwD may adjust their behavior and activities, responding to the experience of the illness (69, 70), and this has been incorporated in models of self-awareness (71). For example, the cognitive awareness model [CAM; (71–73)] suggests a parallel route to process implicit information about ability [see also Piras et al. (74) for an extension of this model and an exploration of self-awareness in people with mild cognitive impairment]. This notion was developed considering clinical observation suggesting that unawareness paradoxically can be accompanied by signs of understanding or representation of difficulties, which are not explicitly expressed (62). This may occur through jokes; symbolic references to dementia-related disabilities, which are not acknowledged; or compliance with treatment and caregiving practices even though such care might not be deemed necessary to the person, due to lack of awareness (62).

Additionally, a number of studies investigating emotional reactivity and behavioral changes in dementia have supported the notion of processes linked to implicit self phenomena. In terms of emotional reactivity, this has been observed, for example, in attentional biases, including heightened implicit reactivity to dementia-related material. Martyr et al. (75) used a modified form of the emotional Stroop including dementia-related words, with bias toward such words indicated by slower responding. Both PwD and their carers showed an increase in response times to salient compared with neutral words. Additionally, in patients, this effect was unrelated to their levels of awareness of condition (75). These findings are indicative of the operation of emotional interference at an automatic unconscious processing level and that awareness may be retained at an implicit level.

Further evidence for an implicit self in dementia has been obtained in a study that explored emotional reactivity to film material in mild AD (25). Participants watched neutral, negative, and positive film clips, with the negative material containing a film about dementia and another about cancer. Despite attenuated responses, reactivity in PwAD was consistent with valence of the stimuli. Critically, higher frequency of negative facial expressions was observed in patients with reduced awareness. This was interpreted as representing implicit self-processes, with unaware PwAD presenting “leakage” of involuntary expressions, and implicit awareness leading to reactivity, which bypasses voluntary control (25). Preserved emotional reactivity to the experience of failure in tasks despite reduced awareness has also been observed in PwAD (26).

In relation to behavioral adaptation, evidence has been produced in both observational studies and experimental settings. Self-imposed driving restrictions despite unawareness difficulties have been observed in PwAD (76), with adaptations such as relying on co-pilots (77) and avoiding driving in unfamiliar environments (78). In the context of research into metacognition, Moulin, Perfect, and Jones found that PwAD allocated their study time appropriately (reducing time for repeated materials) despite an inability to predict performance explicitly in an accurate manner (79). The efficient allocation of study time would indicate that some strategic, but likely unaware, processing was going on. Using estimations of confidence in a forced-choice perceptual task, Geurten et al. (80) report that, although explicit metacognition is impaired in PwAD, implicit introspection may be preserved. Specifically, PwAD asked for cues more often after incorrect than after correct responses, in levels similar to controls, despite limited explicit acknowledgment of poor performance (80). It has also been shown that PwAD persistence in tasks is directly influenced by current performance, with tasks being stopped more often after a sequence of errors (81).

Altogether, these findings suggest implicit affective processing, preferences, and self-knowledge. One issue that has not been clarified, yet, is the extent to which long-term implicit affective preference and behavioral change can be observed. Some studies have suggested that emotional responses linger in PwAD despite loss of declarative memory for the events that caused the emotion. This has been seen in response to films (82) and music (83). In the study by Bomilcar et al., long-term task preference was explored with participants doing tests that had been performed a week before either in a success or failure condition (81). Task preference in the second session was only observed when awareness of performance in the first session was included in statistical models, suggesting initial moments of awareness may boost long-term adaptation even if the content is not available explicitly anymore (81).

### Critical Self

A crucial aspect of our sense of self refers to the explicit records of information we hold about ourselves, i.e., autobiographical memory. When we are asked who we are, typically it is to autobiographical memory that we resort to provide an answer. Memory may be responsible for continuity, one of the core features of the self. It is not surprising, then, that a number of theories have highlighted its role in the maintenance of a sense of self. For example, Conway, following the original philosophical structure of Locke (84), suggests a reciprocal relationship between long-term memory and the self, with memory constraining the structure of the self, and the self-modulating encoding and retrieval of memory (85). Damasio proposes an autobiographical self, made of past memory records as well as future thinking about ourselves, that enriches more basic forms of the self-based on bodily phenomena (18, 86). It is important to highlight that no reified autobiographical self is needed, leading to an infinite regress; the memory records themselves provide a sense of autobiographical self.

Following the Cognitive Awareness Model (CAM) (71–73), lack of awareness is considered a heterogeneous phenomenon, with primary, executive, and mnemonic forms of anosognosia. In conditions such as AD, mnemonic anosognosia may be the main form of unawareness. The model postulates that personal information is consolidated and updated into a storage for personal information [personal data base (PDB)]. Normally, the PDB is continuously updated by experience; however, in PwAD, memory impairments hinder this process. Considering the relationship between self and memory from the perspective of memory changes and loss of self-awareness in dementia, the notion of a “petrified self” has been suggested (4, 87). This term was used, as a metaphor, to highlight two main features of loss of self-awareness linked to memory loss in AD. Firstly, given the damage to medial temporal lobe structures and the resulting pattern of anterograde amnesia, updating of self-concept would be reduced, with PwD incorporating limited new knowledge about themselves. Secondly, remote autobiographical information, particularly semantic memory, which has long been consolidated and acquired hippocampal independence, would support a core identity in PwD. Hence, the metaphor tried to capture the notion of an autobiographical sense of self in PwD, particularly in AD, that was relatively resilient based on personal history, but with less capacity to incorporate new autobiographical knowledge.

In the context of the current article, focus will be given to the latter aspect of that formulation; a nucleus of self-identity formed before the beginning of the condition, which grounds self-concept despite memory loss. It is based on the retrieval of autobiographical information, including recent and remote memories, which is supported by a diffuse network involving different regions such as the hippocampus, and medial prefrontal and parietal cortices (88, 89). This is termed here a critical self, as an analogy with the notion of critical periods of development. It has been shown that autobiographical memory follows a consistent distribution cross-culturally, with features such as a recency effect (better recall of recent information, as observed in other memory processes) and infantile amnesia (85). Another recurrent aspect is the presence of a “reminiscence bump,” with higher recollection of autobiographical memories from adolescence and early adulthood [for a review, Munawar et al. (90)]. Several explanations have been provided for this phenomenon, ranging from the neurobiological [maturation of the frontal lobes; (4)] to the social (life script and identity accounts), which stress an early critical phase of development of the self, which draws on memories around early adulthood (91, 92). It is likely that these two levels of explanations interact.

Evidence for a reminiscence bump in AD has been seen in the form of better recall of remote vs. recent material, with a temporal gradient for episodic and semantic autobiographical memory in this condition (93–96). There are, however, studies that did not report differences in memory retrieval across life periods in AD [e.g., Irish et al. (97)]. Barnabe et al. have suggested that the method used to elicit memories may have an important impact in the presence of a reminiscence bump (98), with the autobiographical interview (99) leading to a reduced temporal gradient due to allocation of fewer memories to a higher number of life periods.

Interestingly, the pattern of autobiographical recollection seems to be reversed for semantic dementia, with better recall of recent as opposed to remote memories (100). This highlights the different neural substrates for memories according to their consolidation status. Most of the evidence seems to indicate that episodic memory relies on the hippocampus, being impaired early in AD and showing a steeper gradient than semantic memory, only affected in later stages of the condition [for a review, see Lenzoni et al. (87)]. This suggests that the critical self in AD is based on remote personal memories, particularly personal semantics, the cognitive store of personal information. It follows that memories from early adulthood are particularly important in the identity of PwAD, with their continued existence (100).

The resilience of this critical self in AD has been investigated in recent studies. El Haj et al. have shown how people with mild AD think about their life stories to maintain continuity in their sense of self, relying more than healthy controls on their autobiographical memory when concerned about potential threats to continuity (101). Research exploring sense of subjective continuity in dementia indicated that most PwD (79%) experience continuity, with those who do not presenting with poorer psychological health (102). Crucially, Tippett et al. suggest that self-continuity may be maintained in AD despite impairments in episodic recollection (that support phenomenological continuity), through the construction of life narratives from semantic memory (semantic continuity) (103). Their findings suggest that better semantic continuity may be particularly relevant for explanations of self-continuity, while confidence in self-persistence may be linked to general and less sophisticated continuity explanations (103).

### Surrogate Self

The surrogate self is composed of mental representations that are not based on neurocognitive systems representing directly the self, being usually elicited through information processing involving perspective-taking abilities. Perspective-taking is a complex and multifaceted phenomenon encompassing social and cognitive aspects and refers to the ability of taking someone else's position or point of view (104). Perspective-taking is understood as an essential ability for our social life, having an important role in the history of our species (104, 105). Indeed, authors defend that perspective-taking is closely related to two important aspects of our social behavior: empathy (106) and theory of mind (107). According to Wilson and Dunn, reflective processing on the self through a third-person perspective may be particularly useful to update beliefs of our own personality (108). In this context, information coming from perspective-taking about the self might influence first-person perspective reflection and, thus, improve self-awareness (109, 110). The surrogate self relies on the cognitive ability of perspective-taking, which is dependent on executive functioning, specifically on switching and inhibition capacities (111). Regarding its neuroanatomical substrates, studies showed that perspective-taking tasks engage a diffuse network including the prefrontal cortex, the temporoparietal junction, and the precuneus [for a review, see Healey and Grossman (106)].

Based on empirical findings, theoretical models have suggested distinct memory systems supporting processing of self- and other-information (71). According to the CAM (71), appraisal of personal information is based on autobiographical memory, generated by individual, social, and cultural experiences and recorded as both experienced events and semanticized self-representations. Other-appraisal, however, relies on general semantic knowledge, stored in different brain systems. It is this generic memory system that can be used for appraisal of others, potentially acting as a surrogate self, allowing people unaware of their difficulties to recognize impairments when exposed to information from an external perspective. Nevertheless, the authors defend that such benefit to self-awareness would depend on the integrity of semantic knowledge and perspective-taking ability (71).

The significance of a surrogate self is seen in the influence of perspective-taking on self-awareness in neurological and psychiatric conditions. A number of studies suggested that self-observation by stroke patients in a third-person perspective leads to increased awareness of motor deficits (112–114). Similar results have been reported for people with psychosis presenting reduced insight (115–117).

In the context of dementia, clinical observations suggest that unaware PwD may be able to acknowledge difficulties similar to their own in others (71). To explore this issue empirically, Clare et al. used a vignette technique as an indirect method to assess awareness in PwD (118). The findings suggested that participants, even when presenting some level of unawareness, were able to correctly identify and give coherent advice for the problem experienced by a third person in the vignette. Additionally, exposure to vignettes helped some of the participants to identify with the situation presented in the vignette spontaneously (118). The use of vignette methodology, but worded in a first-person perspective, has also indicated that PwD make choices comparable with those of controls in relation to advance directives on treatment preferences for life-sustaining interventions [e.g., use of a feeding tube or antiobiotics; (119)]. Preservation of aspects of surrogate self-representation may form the basis for adapted uses of cognitive therapy approaches in people in the early stages of dementia.

The surrogate self in dementia may be affected, however, by neurocognitive impairment. Perspective-taking difficulties have been suggested in the context of dementia, possibly reducing self-awareness (120–122). For example, when assessing the relevance of personality traits adjectives for themselves and for a relative taking a third-person perspective, AD patients showed diminished accuracy of their judgment compared with controls (123). Simm et al. found that cognitive impairments were associated with reductions in reflective self-function, with personal reflective function being influenced by memory difficulties and social reflective function showing an association with processing speed impairments (124). Neuroimaging findings indicate that, when asked to use a third-person perspective, PwAD activate prefrontal regions, potentially relying more on reasoning processes, while older controls recruited visual associative areas, suggesting the use of visual imagery of autobiographical memories (105).

The capacity to use perspective-taking as a way of increasing self-awareness may also be affected by abilities beyond cognition. For example, Mograbi et al. indicated that premorbid personality may be an important factor when evaluating the difficulty of tasks for self and others (125). The authors showed that higher neuroticism and agreeableness were linked to attribution of more difficulty for self/less difficulty for others (125). It has also been suggested that affective components of social cognition are fairly preserved in AD, which may be used to compensate for cognitive components that show impairments [for a review, (126)].

### Extended Self

The concept of extended cognition is characterized by the notion that minds and cognitive systems are grounded on embodied agents interacting with external sources. According to this view, the environment has an active role in driving cognition, which is understood as going beyond brain processes (127). In this context, environmental processes are represented by external sources, as material vehicles of cognition, such as numbers, words, and symbols (128, 129). Further examples of extended cognition include the use of media [such as photos and films; (130)], multi-sensory environmental (131), or evocative objects (132), that allow individuals to remember their past. These include, for example, the PwD home environment, in which long-standing familiarity with objects and architecture may provide support for action.

Departing from this perspective, a notion of extended self can be derived by considering the nature of the relationship between external objects and the self. For example, James suggests that we consider external objects as part of ourselves if they are able to induce affective states (2), whereas McClelland emphasizes agency and sense of control for the incorporation of objects into the self (133). Belk goes beyond the notion of objects being a part of the self, suggesting they also may define a sense of self (134). In this perspective, four levels of extended self are suggested: individual (e.g., personal possessions as jewelry, automobile, make-up, and clothing); family (e.g., residence and furnishings); community (i.e., neighborhood); and group (e.g., social groups) (134).

In this sense, and adopting a cognitive perspective, Heersmink (135, 136) argues that the extended self is related to the autobiographical memory. Indeed, the environment, including objects and people, aids the individual to remember personal memories, which is necessary to create a narrative self. Therefore, we can suggest that the extended self might support and interact with a network involving the hippocampus, and medial prefrontal and parietal cortices (88, 89).

The concept of extended cognition may be particularly relevant for PwD, given the cognitive loss that characterizes the condition. Clark and Chalmers describe a classical thought experiment where Otto, a man with AD, uses a notebook as a memory system (128). The information Otto needs is stored in his notebook and it is accessible for him, as memories “stored” in a properly functioning semantic memory system are, for a person without memory impairments, being similarly available to consciousness and able to guide actions. Otto's notebook is an external artifact integrated in his overall memory system, constituting a central part of his identity and acting as a cognitive agent (128, 129).

From an empirical perspective, a number of studies investigated the role of assistive technology in fostering memory and other abilities in PwD (137), with recent work also summarizing results in relation to electronic devices (138). Findings have indicated that improvements can be seen in various abilities, even in cases of severe dementia [e.g., (139)], but it has been suggested that the focus of the studies needs to be expanded. Specifically, most devices are used to improve ability to undertake tasks, with less attention paid to quality of life, behavioral issues, social connectedness, and recreational activities (138, 139). It has been argued that assistive technologies should be used in PwD to foster social engagement and decision making, which have an important impact on personhood in dementia (140).

Crete-Nishihata et al. investigated directly the influence of technology on the sense of self of PwD (130). Their study explored the impact of personal memory technologies (e.g., multimedia biographies, photos, and home movies) on autobiographical memory retrieval in PwD and mild cognitive impairment. Overall, results showed that these external supports enhanced retrieval of personal past events. Findings also highlighted that the interventions using external sources as memory triggers improved participants' sense of self and reduced apathy (130).

Further evidence from extended self-processes in dementia comes from the literature on awareness of cognitive changes. Cross-cultural studies have explored the role of values and contextual factors in self-appraisal of ability, tapping into community and group levels of the extended self (134). Mograbi et al. showed that PwD in India have lower awareness of their memory problems (141), with a further study suggesting that their relatives also are unaware of difficulties (142). This is line with previous investigations indicating that in this region, dementia and its symptoms are less often understood as a health problem, being more likely to be considered a normal effect of aging by PwD and their caregivers alike (143, 144). This reinforces the notion that the meaning attributed to aging based on external sources (e.g., culture) influences how people are reflecting about themselves, which can be considered a form of extended self.

### Emergent Self

Despite our efforts in categorizing different aspects of the self in dementia, caution should be taken to avoid reifying concepts. There are no selves inside our bodies as things, but rather a variety of processes, caused and constrained by influencing factors ranging from the physiological to the cultural. “Self” is the result of these processes and factors. Within this framework, an important perspective to understand this notion is emergence.

The concept of emergence is based on connectionist models, suggesting that local interactions may lead to properties, states, or processes with a higher level of complexity, to which an identity above and beyond its constituents must be attributed (9). A key notion here is that an emergent feature is greater than the sum of its parts. This theoretical perspective may be particularly relevant to understand processes defined by their binding properties, such as consciousness (145), or, in our case, the self.

In this sense, the emergent self can be understood as a set of unified experiences, which produce higher-order self properties, including holistic phenomenological experiences [see (17) for an example of how intrinsic brain activity may lead to self-agency]. In summary, the “self” may be based on different neural, cognitive, and social processes; but instead of being perceived in a fragmentary manner, it is experienced as a unitary phenomenon. A key question in relation to dementia is how this unitary experience is created and which features of the forms of self previously discussed contribute to this.

According to our framework, selfhood in dementia (and elsewhere) emerges from a number of crucial processes. The embodied self establishes that at our core we are bodies, highlighting the role of bodily experiences and going beyond views of the self that privilege only cognitive or brain processes. The agentic self indicates that this body is driven by action, assuming responsibility for its acts in the environment. The implicit self suggests this body that acts is marked by affective preferences and behavioral responses that may be unknown to us. At a declarative level, the critical self reminds us that this body that acts and has preferences also has a history, possessing a core identity that remains despite brain damage, while the surrogate self reflects the notion that part of our self-knowledge is constructed in interaction with others. The extended self expands the notion of selfhood beyond ourselves; we are also in the external world, in the objects and people that constitute our living environment.

It is from these vital sources that a unified sense of self emerges in PwD. Given the richness of these different self-processes, it is clear that overall this is maintained in dementia, even in later stages of the condition. This topic is of considerable clinical relevance, suggesting that when some processes are impaired, others may compensate for their absence, in a self-regulating system. Similarly, disruption of different aspects of the self may interact to produce emergent properties that characterize the experiences of a PwD. Furthermore, external sources, such as the surrogate and extended selves, that constitute the emergent self in dementia highlight our collective responsibility in supporting selfhood externally when neurodegenerative processes have diminished internal sources of selfhood.

**Figure 1:**
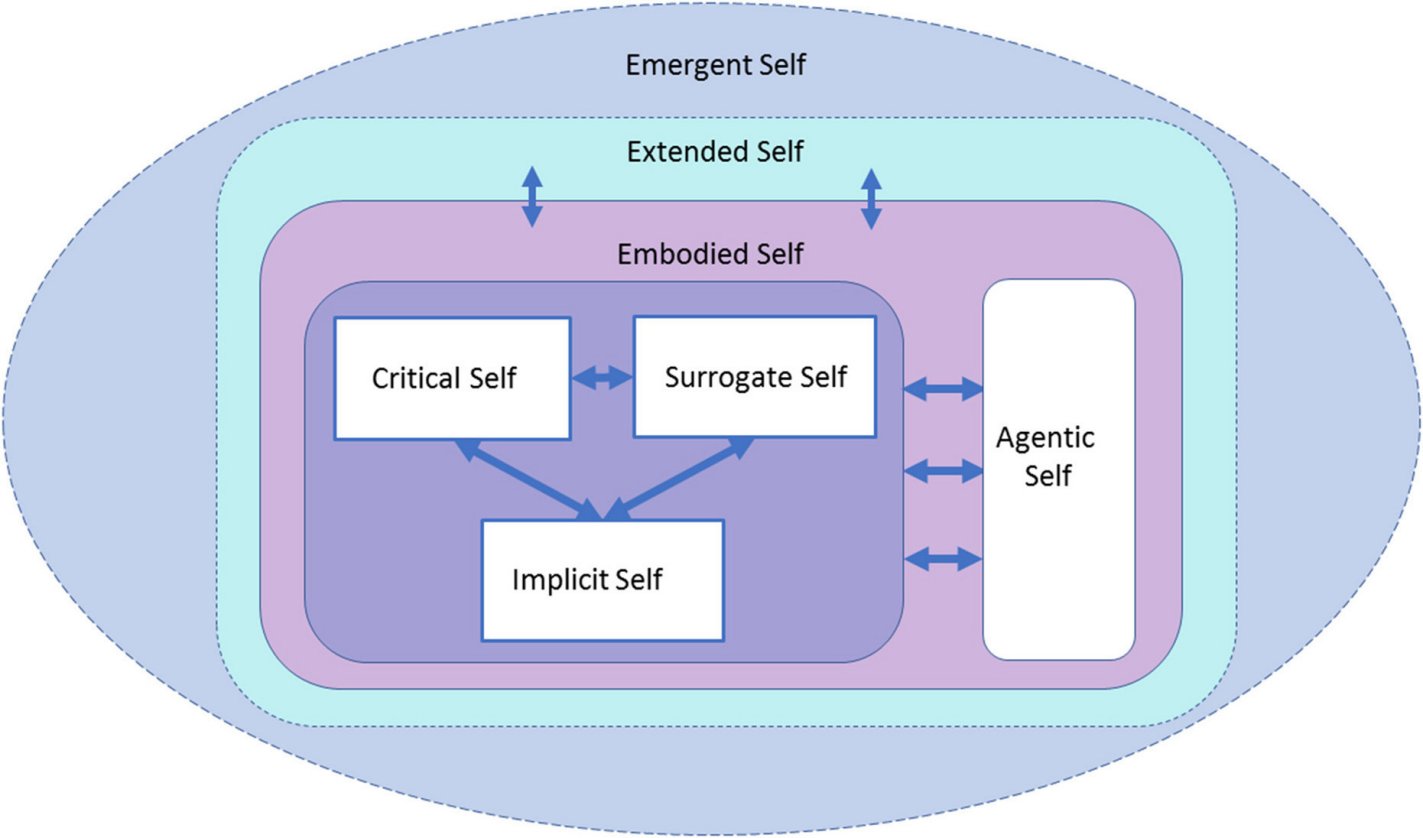
The Components of the Self Model (CoSM). The relationship between the seven forms of self-processes reviewed here is illustrated. Neurocognitive processes, such as the implicit, critical, and surrogate, interact with and are controlled by agentic processes. All these systems are embodied. Beyond an individual perspective, interaction between embodied and social processes leads to extended selfhood. The emergent self is the result of these multiple interactions, providing a unifying context for experience and allowing for some self-processes to compensate for impairments in others.

Table 1 summarizes the seven forms of the self, as discussed in the review, while Figure 1 illustrates the emergent self in dementia.

**Table 1:** The seven selves of dementia.

**Self-processes**	**Definition**	**Examples**	**Evidence in dementia**
Embodied self	Unified experience of the body; awareness and expression of bodily states	• Non-verbal communication • Dancing, drawing, and other forms of corporal expression	Non-verbal communication and corporal expression preserved even in severe dementia, despite impairments in awareness of visceral states (interoception)
Agentic self	Sense of being an agent and influencing life circumstances	• Self-reflection about actions • Decisional capacities in everyday life	Difficulties in decision-making and self-reflective processes, with preservation of agency through emotional and behavioral responses
Implicit self	Non-conscious self-knowledge and preferences	• Use of jokes and symbolic references demonstrating implicit knowledge • Implicit affective and behavioral preferences	Implicit emotional reactivity and behavioral adjustment, but more evidence needed investigating long-term changes
Critical self	A core sense of self-identity and self-continuity	• Remembering past incidents • Factual knowledge about oneself	Self-identity and self-continuity based on remote autobiographical memory from adolescence and early adulthood, especially semantic information
Surrogate self	Self-representations based on third-person perspective information	• Acknowledgment of difficulties when seen in a third-person perspective • Use of vignettes to assist decision making	Some of level of preservation, despite perspective-taking difficulties
Extended self	Incorporation of external objects or existences into the self	• Considering objects as part of ourselves and using external resources to support cognition • Family, community, and group influences in self-awareness	Enhancement of cognitive ability and sense of self, impact of contextual factors in self-appraisal
Emergent self	Emergent unified sense of self	• Feeling of oneness	Phenomenological and ethnographic perspectives suggesting endurance of the self

## Concluding Remarks

The framework used in this paper develops the notion of seven aspects of the self that are pertinent to understanding PwD. These provide a framework that can be incorporated into a model of the self, the Components of the Self Model (CoSM), as shown in Figure 1. Here, the critical, surrogate, and implicit selves are seen as primarily neurocognitive, regulated by the agentic self. These aspects of the self are embodied, including corporealization, and extended though interaction into the environment, producing the emergent self.

The framework and model, as proposed in the current article, may have important implications in terms of clinical management of PwD, also highlighting interesting avenues for future research. For example, considering the role of the body and embodied practices on the self in PwD broadens our horizons in relation to how the condition is represented and experienced. Embodiment allows us to understand the continuity of the self in dementia beyond cognitive impairment, also providing us with a new perspective on caretaking and services. In this view, the body is seen as capable of experiencing and communicating wishes and desires, even in cases of compromised verbal ability (146) and cognitive decline (20, 147–149). The embodied self idea implies that every person with dementia is an individual who expresses themselves with and through a body, affirming the need for person-centered perspectives supportive of personhood in dementia (6).

In relation to the agentic self, excessive focus on self-reflectiveness may have led to the notion of diminished agency in PwD, which have important implications in care for the condition, including in how clinical decision making is allowed and supported. Empowering PwD in relation to their sense of agency is an important treatment direction, fostering their ability to express preferences, take action, and assume responsibility. Here, is important to highlight the crucial role of training caregivers on assisted autonomy, allowing them to distinguish the different stages of dementia and accurately acknowledge PwD functional level (57). This includes formal training to family member to support activity engagement for PwD at home (58). Additionally, future research should explore the sense of agency in dementia using alternative assessment methods, such as those engaging directly with individual intersubjectivity (56) and exploring personal meanings (58).

The notion of an implicit self impacts the assessment and rehabilitation in dementia. Increasing evidence suggests that implicit processes are more complex in nature than previously thought (62), indicating the need to consider this dimension in the interaction with PwD. According to this perspective, implicit awareness may extend to complex stimuli, for example, in the case of social judgments and decision making (62), impacting everyday life of PwD. From a clinical perspective, considering assessment, this highlights how tailoring it may prevent excessive negative emotional responses or exposure to experiences that may be aversive in nature. Regarding rehabilitation, paradigms relying on implicit abilities may prove particularly useful for dementia (150). In both cases, paying attention to the implicit preferences, knowledge, and non-verbal behavior of PwD is particularly important. Research priorities include testing the efficacy of these new care paradigms and understanding better long-term implicit behavioral changes in dementia.

Findings in relation to the critical self indicate that core aspects of identity are maintained, which may support selfhood in dementia, while also pointing to potential interventions. For example, this would include use of reminiscence therapy, where individualized approaches take into account the past history of a person. However, this needs to be done sensitively, balancing with the need to expose PwD to new information and associations (151). Further research into memory consolidation may elucidate which elements acquire hippocampal independence sooner, with neuroimaging studies helping to predict the individual profiles that may struggle with declarative aspects of identity. Future studies should also consider how autobiographical memory interacts with other forms of self-processes in dementia, for example, with implicit preferences (e.g., music) and motor habits instantiated in procedural memory (e.g., dancing).

The presence of surrogate self-processes can be used as a therapeutic tool in dementia. For instance, exposure of self-information in a third-person perspective may be used to foster self-awareness and improve other clinical outcomes. Unawareness has been linked to risk-taking, reduced treatment compliance, and early institutionalization, in addition to higher levels of caregiver distress and burden (152–155), so interventions to mitigate this, if done sensitively, may have important clinical consequences. Additionally, preserved general semantic knowledge may serve as an additional source to support treatment decisions. For instance, the use of vignettes can be expanded in clinical settings, as a way of providing additional information and assisting in decision making. For the development of these tools, however, a better understanding of potential impairments of PwD in social cognition is needed, exploring in deep how perspective-taking, and related abilities, such as theory of mind and empathy, are affected in the condition.

The way external objects are incorporated into self-processing needs to be investigated further in dementia. Empirical studies demonstrated the beneficial influence of external objects in improving autobiographical memory retrieval in PwD by the use of multimedia tools (130) and external environmental stimuli (131). Although the notions behind extended cognition are routinely incorporated in neuropsychological rehabilitation, for example, in the use of compensatory strategies, future studies should explore how these external aids impact selfhood. Attention should also be paid to external sources that constitute the self in dementia, preventing unnecessary losses that may weaken self-identify (e.g., leaving their own house to reside in nursing homes) (129, 135). Additionally, as suggested above, the focus of current studies should be expanded to other factors with a likely impact on personhood in dementia, such as social connectedness and agency (137, 138, 140). Higher levels of the extended self, linked to community and group processes, highlight the need for programs that foster social awareness about dementia.

Finally, the notion of an emergent self emphasizes how promoting compensation and continuity are two important directions in helping PwD retain their sense of identity. Allowing space for multiple forms of the self to express themselves, through bodily, agentic, implicit, and identity processes, as well as striving to compensate losses with surrogate and extended processes, is essential. Needless to say, the perspective suggested here is not that there are only seven forms of the self in dementia. On the contrary, we highlight that for each person with dementia, there are multiple sources that feed into these processes. It is from the combination of these multiple selves, and others beyond, that the uniqueness of each individual emerges, facilitating a person-centered approach.

## Author Contributions

IB, EB, RM, and DM contributed writing and revising the manuscript. All authors contributed to the article and approved the submitted version.

## Conflict of Interest

The authors declare that the research was conducted in the absence of any commercial or financial relationships that could be construed as a potential conflict of interest.
